# Alternative Solvents for Pectin Extraction: Effects of Extraction Agents on Pectin Structural Characteristics and Functional Properties

**DOI:** 10.3390/foods14152644

**Published:** 2025-07-28

**Authors:** Alisa Pattarapisitporn, Seiji Noma

**Affiliations:** 1 The United Graduate School of Agricultural Sciences, Kagoshima University, 1-21-24 Korimoto, Kagoshima 890-0065, Japan; alisa.pattara@gmail.com; 2 Faculty of Agriculture, College of Natural Sciences, Institute of Education and Research, Saga University, 1 Honjo, Saga 840-8502, Japan

**Keywords:** pectin extraction, chelating agents, subcritical fluid, deep eutectic solvents

## Abstract

Pectin is a multifunctional polysaccharide whose structural attributes, including degree of esterification (DE), molecular weight (MW), and branching, directly affect its gelling, emulsifying, and bioactive properties. Conventional pectin extraction relies on acid- or alkali-based methods that degrade the pectin structure, generate chemical waste, and alter its physicochemical and functional properties. Although novel methods such as ultrasound-assisted extraction (UAE), microwave-assisted extraction (MAE), and enzyme-assisted extraction (EAE) are recognized as environmentally friendly alternatives, they frequently use acids or alkalis as solvents. This review focuses on pectin extraction methods that do not involve acidic or alkaline solvents such as chelating agents, super/subcritical water, and deep eutectic solvents (DESs) composed of neutral components. This review also discusses how these alternative extraction methods can preserve or modify the key structural features of pectin, thereby influencing its monosaccharide composition, molecular conformation, and interactions with other biopolymers. Furthermore, the influence of these structural variations on the rheological properties, gelling behaviors, and potential applications of pectin in the food, pharmaceutical, and biomedical fields are discussed. This review provides insights into alternative strategies for obtaining structurally intact and functionally diverse pectin by examining the relationship between the extraction conditions and pectin functionality.

## 1. Introduction

Pectin is a complex heteropolysaccharide primarily found in the primary cell walls and middle lamellae of higher plants, where it plays a key structural role by providing cohesion and flexibility to plant tissues [1]. It is especially abundant in citrus peels, apple pomace, sugar beet pulp, and various tropical fruit residues, which are commonly used as commercial sources because of their high pectin yield and availability as agro-industrial byproducts [2]. The structural diversity and complexity of pectin contribute to its wide range of functional properties, including gelling, thickening, stabilizing, and emulsifying behaviors, making it a valuable hydrocolloid in both food and non-food industries [3,4]. The inherent structural adaptability of pectin, whether maintained in its native form or modified through selective and innovative extraction techniques, has enabled the precise tailoring of its physicochemical and functional properties for specific applications, as its multifunctionality is fundamentally attributed to the complexity of its molecular architecture [5]. Thus, advancing the understanding and control of this structure through modern extraction and modification strategies offers significant potential for expanding its high-value, sustainable, and health-promoting applications in food, pharmaceutical, and biomedical fields.

Conventional pectin extraction methods typically rely on acid hydrolysis using mineral or organic acids under high temperatures (typically 80–100 °C) and controlled pH conditions, usually between 1.5 and 3.0 [6,7,8]. This process promotes the solubilization of pectin from the plant cell wall matrix by breaking down the plant cell wall structure and disrupting the bonds between pectin and other polysaccharides, thereby allowing the efficient release of pectin [9]. However, despite its industrial efficiency, this approach has several significant limitations that have prompted interest in alternative methods. One of the primary drawbacks of acid-based extraction is pectin structural degradation. Harsh conditions can lead to partial depolymerization and demethylation, thus resulting in pectin with reduced molecular weight (MW) and inconsistent functional properties [10,11]. Furthermore, this process often damages the degree of esterification (DE), rhamnogalacturonan regions, and side chains, which are important for physicochemical and biological functions [12,13]. This compromises the structural integrity and limits the potential use of pectin in high-value applications such as pharmaceuticals and nutraceuticals. Numerous review articles have extensively covered the physical technologies for pectin recovery, such as conventional thermal extraction, ohmic heating, microwave-assisted extraction (MAE), and ultrasound-assisted extraction (UAE). Heating techniques have demonstrated the ability to enhance extraction efficiency by accelerating cell wall disruption and improving mass transfer [14,15]. However, they are often used in conjunction with acidic or alkaline solvents that can degrade the structural integrity of pectin [8,16,17]. In contrast, alternative solvent-based approaches offer gentler and more selective methods for extracting and preserving pectin structures. This article focuses specifically on pectin extraction agents with an emphasis on alternative solvent systems [18,19,20]. The potential of alternative solvents involves chelating agents that induce pectin release by binding to metal ions, particularly calcium, which is crucial for the stability of plant cell walls [21]. These methods minimize hydrolysis and allow for greater retention of the rhamnogalacturonan domains, which are often degraded by acid or alkali treatments [22]. Other novel solvents include supercritical and subcritical fluids and deep eutectic solvents. These media systems not only preserve the functional groups of pectin but also reduce the environmental impact associated with wastewater treatment and chemical hazards [23,24,25,26].

By shifting the focus from thermal techniques to solvent composition and extraction environment, this study aims to provide a detailed understanding of how alternative solvents can improve the quality and sustainability of pectin. These approaches are aligned with the growing demand for clean-label ingredients and green processing technologies, particularly for high-value applications in the food, pharmaceutical, and biomedical sectors.

## 2. Pectin

**Figure 1:**
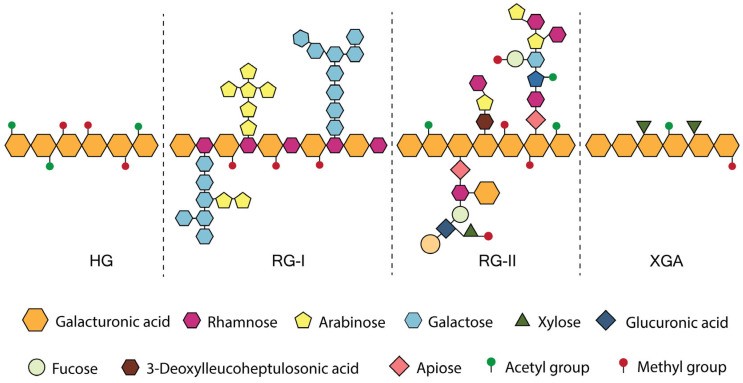
Properties of the pectin molecular structure, highlighting the main domains: homogalacturonan (HG), branched rhamnogalacturonan I (RG-I), rhamnogalacturonan II (RG-II), and xylogalacturonan (XGA) domains [27].

Pectin is a dietary fiber found in fruit and vegetable cells. The pectin structure contains 100–1000 units of galacturonic acid as the backbone, which can be replaced by other saccharides in the side chain. Pectin generally contains at least 65% galacturonic acid; however, this proportion can vary depending on the degree of neutral sugar side chain substitutions along the pectin backbone. As shown in Figure 1, the pectin structure is divided into three domains: HG regions, branched RG-I, RG-II, and XGA domains, depending on the type of saccharide contained [27,28]. Pectin typically comprises approximately 65% of HG, whereas RG-I and RG-II constitute the remaining 20–35% and <10%, respectively [29]. HG was found in pectin extracted from apples, beets, and citrus using 0.1 M HCl at 80 °C for up to 72 h [30]. Most of the neutral sugars and a small amount of galacturonic acid were solubilized during the process. The link between uronic acid residues was relatively stable at pH < 2. However, this preferentially cleaves the links between galacturonic acid and neutral sugars in the side chain [30]. RG-I is the only pectin not entirely composed of a linear galacturonic backbone. Instead, it is a branching polymer with a repeating structure of α-1,4-D-galacturonic acid and α-1,2-L-rhamnose. The side chains of ß-1,4-galactan, branched arabinan, and/or arabinogalactan can be substituted by rhamnose residues in the backbone [31]. RG-II has a galacturonic acid backbone similar to that of HG; however, the backbone is extensively branched because the side chains are substituted. Different types of neutral sugars may be present in the side chains, including arabinose, apiose, fucose, galactose, rhamnose, acetic acid, glucuronic acid, galacturonic acid, xylose, and fucose, which are linked to the formation of short or long chains attached to the carbon atoms of the backbone [32]. XGA is characterized by the substitution of the galacturonic acid backbone with xylose residues, typically at the C-2 or C-3 positions, which imparts unique properties to the polymer [33].

**Figure 2:**
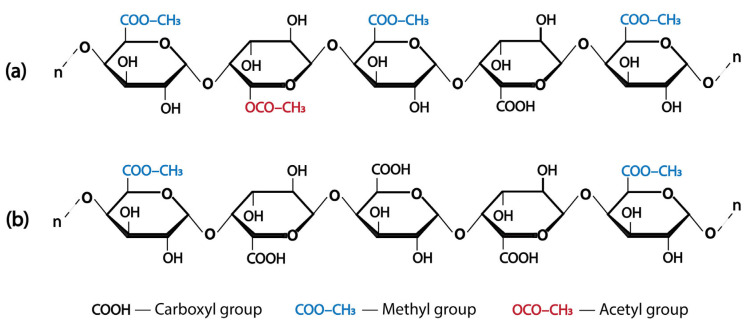
The chemical structure of (**a**) high-methoxyl pectin (HMP) and (**b**) low-methoxyl pectin (LMP) [34].

Pectin can be classified based on its DE, which refers to the proportion of galacturonic acid residues in the pectin backbone esterified with methanol. DE refers to the natural esterification status of the galacturonic acid residues in plant cell walls; however, DE can become altered during extraction. Based on DE, pectin is generally divided into two main types: high-methoxyl pectin (HMP) and low-methoxyl pectin (LMP) (Figure 2) [34]. HMP is characterized by a DE greater than or equal to 50%, indicating that at least half of its carboxyl groups are esterified. In contrast, LMP contained more than 50% free carboxyl groups, corresponding to a DE of < 50% [35,36].

This distinction in esterification significantly influences their gelling behavior. HMP typically forms gels under acidic conditions (pH 2.5–3.5) in the presence of high concentrations of soluble solids such as sucrose or dextrose and does not require the addition of divalent ions [37]. In contrast, LMP forms gels through ionic cross-linking with calcium or other divalent cations, making them suitable for low-sugar or sugar-free applications [38]. The source and type of raw material significantly influenced pectin yield and composition. Pectin content varies considerably among different plant sources, typically ranging 25–35% (*w*/*w*) in citrus peels, 15–20% in apple pomace, 15–30% in sugar beet pulp, and 5–25% in sunflower heads [2,39].

## 3. Conventional Pectin Extraction

**Figure 3:**
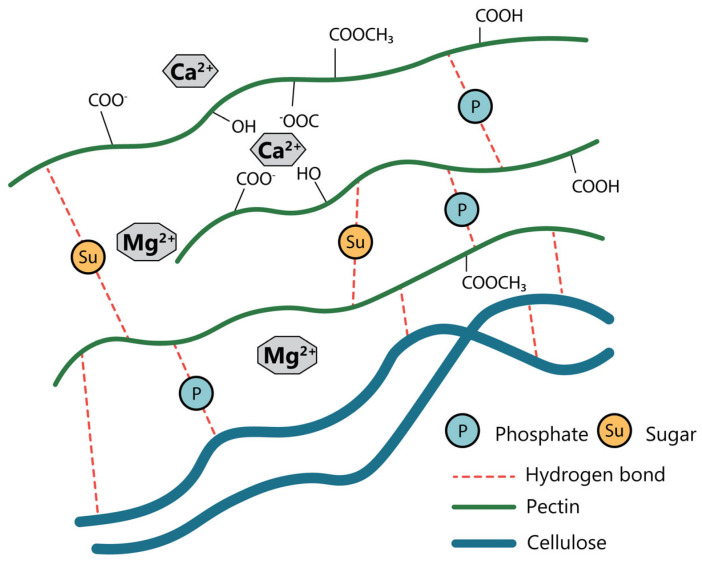
The ideal model of protopectin structure, redrawn with permission from Ueno et al. [51].

The conventional method for pectin extraction, widely implemented at the industrial scale, involves acid hydrolysis using dilute mineral acids, such as hydrochloric or nitric acid, at elevated temperatures, typically ranging from 80 to 100 °C, followed by alcohol precipitation to recover the solubilized pectin [40,41]. Alternatively, weaker organic acids such as citric or acetic acid can be used. Although mineral acids offer a stronger hydrolytic action and higher extraction efficiency, organic acids provide a gentler extraction environment, which may better preserve the structural integrity of the extracted pectin [26,42,43]. Under strongly acidic and thermal conditions, the glycosidic bond-anchoring pectin within the plant cell wall matrix is cleaved, leading to the depolymerization of insoluble protopectin into its soluble form, which is subsequently released into the extraction liquor [44]. However, if the conditions are excessively harsh or prolonged, further hydrolysis may occur, leading to significant degradation of the solubilized pectin and a reduction in its MW [45]. Protopectin, a water-insoluble precursor of pectin, is a partially methylated complex that becomes water soluble upon hydrolysis (Figure 3) [46]. This schematic depicts the intricate network formed by pectin chains, cellulose microfibrils, and divalent cations such as Ca^2+^ and Mg^2+^. Pectin predominantly exists as a polymer of galacturonic acid residues, which can be partially methyl-esterified and acetylated to form long-chain polysaccharides. These chains are often cross-linked via ionic bridges created by divalent cations between the carboxyl groups of adjacent galacturonic acid units (see points 1–4), thereby contributing to the insolubility of protopectin and the rigidity of the plant cell wall structure [47,48,49]. During extraction, the initial acid treatment facilitated the release of pectin from the plant tissue. In addition to hydrolyzing protopectin, acid treatment plays a crucial role in dissolving the calcium and magnesium cross-links that stabilize the pectin–cellulose complex, thereby enhancing cell wall disintegration and improving extraction efficiency. This results in a high yield of pectic polysaccharides, particularly those rich in homogalacturonan regions, which dominate the solubilized fraction [50].

Alkaline extraction techniques often employ sodium hydroxide, potassium hydroxide, or aqueous ammonia. The operations are normally conducted under regulated alkaline conditions at moderate temperatures (75–95 °C), at pH 8.5–13 [52,53,54]. Alkaline agents break ester bonds and disrupt hydrogen bonding between pectin and other structural components, such as cellulose and hemicellulose, thereby facilitating the release of pectic polysaccharides into the solution [55]. This method facilitates the recovery of pectin with a low DE and distinct functional characteristics, unlike acidic extraction, which typically yields HMP [56]. Alkaline treatment frequently leads to a substantial reduction in the DE owing to the saponification of methyl esters [57]. This can be advantageous for producing LMP, which forms a gel in the presence of divalent cations such as calcium, making them suitable for specific industrial applications such as reduced-sugar or calcium-set food products [58]. Furthermore, the MW of alkaline-extracted pectin tends to be lower due to degradation mechanisms such as β-elimination [53,59]. The strong de-esterifying and hydrolytic nature of alkaline solutions may also result in the partial degradation of the homogalacturonan backbone, leading to a reduced MW and, in some cases, diminished gelling and emulsifying capabilities.

Nevertheless, both acidic and alkaline extraction processes, while effective, have drawbacks, including high energy consumption, degradation of heat-sensitive components, and generation of chemically intensive effluents [52,60,61]. These conditions can cause depolymerization, reduce MW, and decrease DE. Additionally, side chains, such as arabinose and galactose, in the rhamnogalacturonan regions may be lost, thus compromising the physicochemical and functional properties of the extracted pectin. Hence, pectin extraction techniques have advanced considerably, shifting from conventional methods to environmentally conscious approaches designed to optimize the yield, structural preservation, and functionality.

## 4. Alternative Solvents for Pectin Extraction

Solvent extraction methods have emerged as viable alternatives to traditional acid hydrolysis for pectin recovery, offering environmentally friendly strategies that allow greater control over the structural properties of extracted biopolymers [62]. Despite these advantages, several limitations remain, including comparatively low extraction yields and the occasional formation of undesirable byproducts, which pose challenges for industrial-scale implementation [63]. However, alternative solvent extraction methods, including chelating agents, super/subcritical extractions, and DESs, continue to gain attention as promising techniques for achieving sustainable pectin production.

### 4.1. Chelating Agents

**Figure 4:**
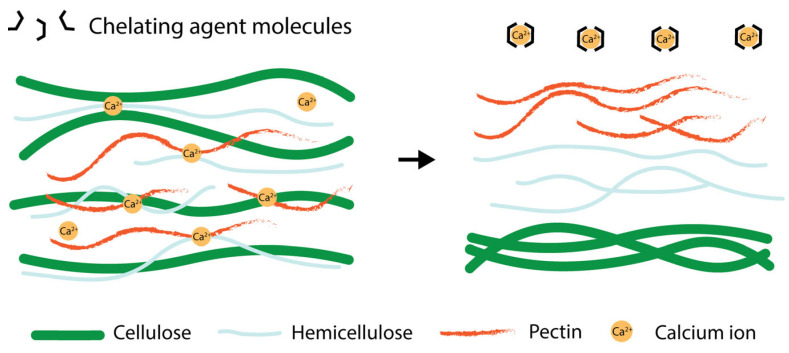
The effect of the chelating agent on pectin extraction. The arrow indicates the action of chelating agents removing calcium ions and facilitating the release of pectin from the cell wall matrix.

Chelating agents have garnered increasing attention as alternative solvents for pectin extraction because of their unique ability to disrupt the ionic interactions between pectin chains and divalent metal ions (Ca^2+^ and Mg^2+^), which play a critical role in maintaining the structural integrity of plant cell walls. In plant tissues, pectin exists in the form of protopectin, a complex of pectic polysaccharides tightly bound to the cell wall matrix through calcium-mediated cross-linking (Figure 4) [21,22]. Chelating agents selectively bind to and sequester these divalent cations, weakening the matrix and facilitating pectin solubilization without requiring the extreme pH or high temperatures associated with conventional acid or alkali extraction methods [64]. Various chelating agents have been investigated for pectin extraction, each differing in metal ion affinity, extraction efficiency, and impact on pectin quality. The most widely studied compounds are cyclohexanediaminetetraacetic acid (CDTA), ethylenediaminetetraacetic acid (EDTA), sodium hexametaphosphate (SHMP), ammonium oxalate, oxalic acid, and citric acid [65]. In contrast to strong mineral acids, chelating agents are generally prepared at near-neutral to mildly acidic pH values, consequently maintaining the essential pectin characteristics [30,66]. Nevertheless, chelating agents can facilitate enzymatic pectin extraction by disrupting calcium-mediated cross-links such as egg-box structures in homogalacturonan regions. This loosening of the pectin network enhances the accessibility of enzymes, thereby improving the efficiency of enzymatic extraction [67]. The extraction and production of pectin using chelating agents are listed in Table 1.

EDTA facilitated the extraction of calcium-bound pectin under near-neutral conditions, yielding pectin with superior MW and improved structural integrity relative to acid-extracted pectin. According to a previous study, pectin extracted from orange peels using 0.5% EDTA and ammonium oxalate at 90 °C for 90 min had an average MW of approximately 102 and 91.4 kDa, respectively, which is significantly higher than that extracted using HCl (84.5 kDa) [68]. Zhang et al. [69] found that EDTA-extracted pectin from sweet potato residues had a significantly higher MW than pectin extracted with acids or alkalis. However, pectin extracted using NaOH, which was characterized by the lowest MW and highest branching, showed the strongest anti-inflammatory activity, whereas the activity declined progressively with decreasing levels of branching. CDTA has been successfully applied to raw materials such as apple pomace and sugar beet pulp, yielding pectin at rates of 31.9% and 27.5%, with DE values of 74.0% and 55.0%, respectively [21]. Apple pomace and sugar beet pulp were extracted using the same solvent. However, their structures differed, indicating that the pectin structure was naturally influenced by its origin. Apple pomace comprises HMP with a high MW, whereas sugar beet pulp has lower DE, MW, and highly branched sugars. SHMP has also been employed to extract pectin from hawthorn pomace and prickly pear peel, yielding moderate recovery with high galacturonic acid contents ranging from 68.94% to 72.6%, respectively [70,71]. However, a limitation of SHMP is its potential as residual phosphate, which can contribute to increased ash content and compromise the purity of the final product if not adequately removed during processing [24]. Citric acid, a mild organic acid, can function as both a proton donor and a chelating agent by forming stable complexes with calcium. It is considered a natural and food-grade solvent, which makes it attractive for environmentally friendly processing. Citric acid has been used to extract HMP from mango and orange peels, with yields ranging from 13.9% to 17.9%, DE values between 48% and 88%, and high MW, indicating minimal degradation of the pectin backbone [22,72,73]. Wang et al. [72] reported that pectin extracted from mango peels using citric acid exhibited a very high MW (2320 and 2858 kDa) and maintained 100% emulsion stability against creaming after one week of storage at 80 °C. In contrast, pectin with a lower MW (272–665 kDa) exhibited a significant decline in emulsion stability, with a reduction of up to 40%. Pectin extraction using citric acid and sodium citrate buffers resulted in moderate yields with DE values of 34% and 47.7%, respectively. While citric acid yielded a higher MW (314 kDa), sodium citrate yielded a lower MW (204 kDa), indicating that the buffer conditions may affect the polymer size. Ammonium oxalate provided the highest yield (14.8%) and galacturonic acid content (71.7%), along with a relatively high MW (336.8 kDa) and low DE (31%), suggesting effective structural preservation. In contrast, alkaline extraction with NaOH led to severe de-esterification (DE 4%) and reduced MW (239 kDa) despite a comparable yield (13.7%). These findings suggest that citric/citrate solvents can balance the structural integrity and yield, while ammonium oxalate preserves the pectin structure better than alkaline conditions [74]. Oxalate anions have a high affinity for calcium and generate insoluble calcium oxalate complexes that efficiently break down protopectin structures. In another study, pectin extraction from sunflower heads using 0.76% ammonium oxalate at 85 °C for 90 min resulted in a yield of 7.36% [24]. A combination of ammonium oxalate and oxalic acid applied to tomato processing waste resulted in a markedly higher yield of 34.6%, producing HMP with a DE of 88.98%, suggesting effective chelation and minimal demethylation under the applied conditions [75].

In previous studies, chelating agents have demonstrated significant potential for pectin extraction, yielding pectin with diverse structural characteristics, depending on the raw material, solvent composition, and extraction conditions. High pectin yields were obtained using CDTA, ammonium oxalate, and EDTA as chelating agents, whereas citric acid afforded a moderate yield. However, the chemical structure of the extracted pectin varied depending on the type of chelating agent used, thus suggesting that each method can be selectively applied according to the intended functional applications in future studies. High-DE pectins have consistently been obtained using ammonium oxalate and citric acid, whereas pectins with high MW have been reported following extraction with EDTA, ammonium oxalate, and citric acid. In contrast, alkaline extraction significantly reduced both the DE and MW, resulting in structurally degraded pectins, whereas conventional acid extraction yielded pectins with moderately preserved structural integrity. Notably, both citric acid and ammonium oxalate have been shown to preserve DE and MW, with citric acid promoting a higher degree of branching (DB). These findings emphasize the potential of chelating agents in producing pectin with desirable physicochemical properties while minimizing its degradation, thus offering a promising approach for sustainable and efficient pectin extraction.

**Table 1:** Overview of the yield and physicochemical properties of pectin extracted using chelating agents.

Materials	Solvents	Extraction Conditions	Yield	Product Structures				Functionality	Ref.
				GalA (%)	DE (%)	MW (kDa)	Branching		
Orange peel	* HCl, pH 2.5	90 °C for 90 min in water bath	29.6	-	-	84.5	-	-	[68]
	Ammonium oxalate, 0.25%		30.1			91.4			
	EDTA, 0.5%		27.7			102			
Sweet potato residue	* NaOH, pH 11	SLR 1:25 for 90 min at 80 °C using conventional heating, 2 times	3.16	38.3	0.14	44.5	HG 20.5%, RG-I 75.4%	Highest anti-inflammation (1st)	[69]
	* HCl, pH 3.0		2.14	54.1	14.3	69.8	HG 43.1%, RG-I 46.9%	Anti-inflammation (4th)	
	Water		2.32	41.5	47.5	58.4	HG 37.1%, RG-I 63.2%	Anti-inflammation (2nd)	
	EDTA, 1%		2.17	50.8	10.6	71.3	HG 36.57% and RG-I 55.24%	Anti-inflammation (3rd)	
Apple	CDTA, pH 6.5	Extraction at 80 °C for 30 min	31.9	49.2	74.0	high MW	-	-	[21]
Sugar beet			27.5	48.4	55.0	low MW	-	-	
Prickly pear peel	SHMP, 0.75%	Using ohmic heating process with field strength of 12.5 V/cm at 90 °C for 45 min	3.32	72.6	21.2	-	-	-	[71]
Mango peel	Citric acid, pH 2.5	SLR 1:40, for 2 h at 80 °C using conventional heating	17.9	Sugar content 55.9%	66.5	up to 904	Highly branched	-	[22]
Mango peel	Citric acid, pH 2.5	SLR 1:40, for 15 min at 80 °C using conventional heating	16.7	52.2	86.8	2858	-	Emulsion	[72]
	Citric acid, pH 2.5	SLR 1:40, for 15 min at 80 °C using UAE	17.2	53.4	86.8	2320	-		
Orange peel	Citric acid, pH 2.41	Under thermal process at 86.36 °C for 64.85 min	13.9	AUA 77.3%	48.2	EW 2380	-	-	[73]
Chicory root	* NaOH, pH 12	SLR 1:20 for 1 h at 85 °C using conventional heating	13.7	57.5	4	239	DB 1.3	Sensitive to calcium ion, rigid gel	[74]
	Citric acid, pH 2		13.3	66.2	34	314	DB 3.4	Thickening property	
	Sodium citrate, 0.5%		8.8	63.7	47.7	204	DB 1.0	Weak syneresis	
	Ammonium oxalate, 0.5%		14.8	71.7	31	337	DB 0.7	Superior hardness, rigid gel	
Sunflower head	Ammonium oxalate, 0.76%	Extraction time 1.34 h, SLR 1:15	7.36	76.2	39.2	316	-	Antioxidant	[24]
Tomato waste	Ammonium oxalate/oxalic acid, pH 3.26	Extraction time 15+15 min at 80 °C with 37 kHz UAE	34.6	AUA 57.16%	89.0	-	-	-	[75]

* Conventional solvent; AUA, Anhydroronic acid; GalA: Galacturonic acid; SLR: Solid–liquid ratio (g/mL); DB: Degree of branching; EW: Equivalent weight.

### 4.2. Subcritical Fluid Extraction

**Figure 5:**
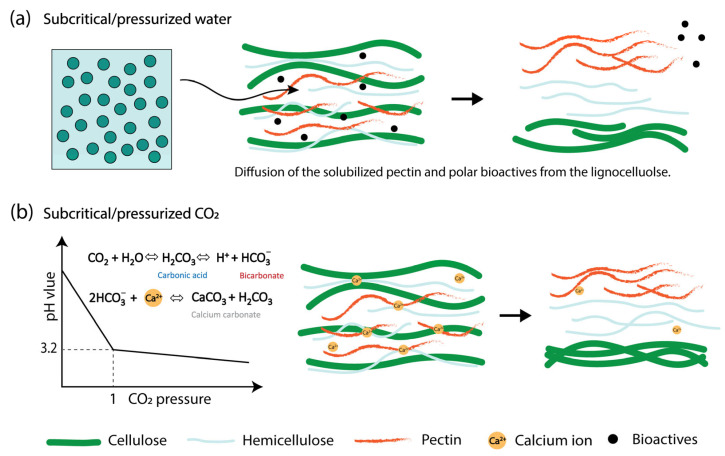
Effects of subcritical (**a**) water and (**b**) CO_2_ on pectin extraction. Arrows represent the release of pectin and other components from the cell wall matrix.

Subcritical fluid extraction has emerged as an environmentally friendly alternative to conventional pectin extraction techniques [76]. These methods utilize water or carbon dioxide (CO_2_) under specific pressure and temperature conditions to enhance the extraction efficiency while minimizing chemical use and structural degradation of the target compound. Pectin extraction using subcritical water, typically at 120–200 °C and 1–5 MPa, is preferred to avoid excessive thermal degradation [77,78]. Under subcritical conditions, the ionic product of water increases and its dielectric constant decreases, thereby augmenting its ability to solubilize polar molecules and hydrolyze plant cell walls (Figure 5a) [79]. Subcritical water acts as a hydrolytic medium that breaks down the plant matrix and facilitates the release of pectin-rich fractions [51,80]. Alternatively, CO_2_ can dissolve in the water present in biomass, forming carbonic acid in situ under pressure (Figure 5b). This weak acid aids in the mild hydrolysis of protopectin and calcium cross-links, enabling pectin solubilization without the need for added mineral acids [77,81]. Subcritical water is proficient at extracting polar molecules and partially hydrolyzing lignocellulosic materials [82]. Various subcritical fluid extraction strategies have been explored for pectin recovery from fruit-processing byproducts, as shown in Table 2.

Subcritical water extraction has demonstrated promising efficiency, with citrus peel and apple pomace yielding 22.0% and 16.7% of pectin, respectively. These extracts were classified as HMP with DE exceeding 74.7–86.0% and MW ranging from 53.4 to 69.5 kDa. In addition, citrus pectin, with higher galacturonic acid content and MW but lower DE, demonstrated greater antitumor activity, whereas apple pectin, characterized by higher DE and DB, exhibited stronger antioxidant activity [23]. Modifying the subcritical conditions by incorporating 0.4% SHMP and 50 mM HCl significantly increased the yield from yuzu flavedo to 15.59% compared to the application of subcritical water, which yielded only 0.95% [51]. Supercritical and pressurized CO_2_-based methods have also been applied, with varying outcomes depending on the matrix and conditions. Pressurized CO_2_ extracted 3.8% pectin from satsuma mandarin peels, characterized by a remarkably high DE (>90%) and MW of 85 kDa, indicating minimal structural degradation and enhanced branching [77]. Acid-based extractions using citric acid (pH 1.4–3) and (2.5) yielded pectins with broader variability in structural characteristics. Citric acid extraction at pH 1.4 from Satsuma mandarin peel resulted in yields of up to 18%, with DE values exceeding 69% and MWs up to 1674 kDa [83]. However, HCl extraction under similar thermal conditions produced a lower-MW pectin (65 kDa), potentially owing to stronger depolymerization effects [77]. Notably, high hydrostatic pressure combined with citric acid treatment (500 MPa for 10 min) enhanced both the extraction efficiency and molecular integrity, resulting in a 19% yield. Despite differences in extraction methods, all pectins extracted from Satsuma mandarin peels exhibited high MW (1201–1871 kDa) and excellent emulsifying stability, maintaining 100% stability after 30 days of storage at 4 °C [83]. Extraction of pectin from cocoa pod husks using subcritical water (120 °C, 10 min) yielded 6.58% LMP with a higher DE (37.87%) and AUA (66.5%) compared to citric acid (pH 3), which produced only 3.2% yield with lower DE (29.8%) and AUA (17.5%). The significantly lower EW of the subcritical water extract suggests more effective solubilization and partial hydrolysis, highlighting its superior efficiency and structural recovery compared to those of the mild acid extraction [84]. Jafarzadeh-Moghaddam et al. [85] also reported that subcritical water extraction resulted in a reduction in the MW of pectin while preserving a higher galacturonic acid content and greater DE than conventional acid extraction. Citric acid extraction under pressurized water with added CO_2_ retained the MW of pectin (149 kDa) more effectively than extraction under atmospheric conditions (127 kDa) or pressurized water alone (68 kDa) [86].

The findings from various studies suggest that subcritical water extraction is effective in maintaining the DE of pectin, although it often results in a reduced MW compared to the conventional acid method. In contrast, subcritical CO_2_ extraction improved the DE, MW, and RG-I structures. Additionally, the use of co-solvents in combination with subcritical fluids has been shown to enhance the pectin yield. These data demonstrate that both subcritical water and CO_2_ extraction provide ecologically friendly options while preserving or enhancing the essential pectin characteristics.

### 4.3. Supercritical Fluid Extraction

Supercritical fluid extraction involves heating a fluid, commonly water or CO_2_, beyond its critical temperature and pressure (374 °C and 22.1 MPa for water; 31.1 °C and 7.38 MPa for CO_2_), where it exhibits unique physicochemical properties [78,87]. In this state, the fluid exhibits gas-like diffusivity and liquid-like solvating power, enabling it to efficiently penetrate the porous biomass and dissolve a wide range of target compounds. These properties make supercritical fluid extraction a highly tunable and environmentally friendly extraction method because it eliminates the need for organic solvents and enables selective extraction through temperature and pressure adjustments [88]. Although supercritical fluid extraction is an emerging technology for pectin recovery, it is less frequently applied for the direct extraction of pectin than subcritical fluid methods [89]. A major limitation is the nature of pectin, a high-MW hydrophilic polysaccharide that is not readily soluble or diffusible under supercritical conditions, particularly in the absence of suitable co-solvents [51,90]. Instead, they are commonly used to extract bioactive compounds associated with pectin. This is because supercritical fluids are particularly effective for extracting non-polar and moderately polar compounds, such as lipids, essential oils, and bioactive phytochemicals, offering high efficiency and selectivity [91,92].

Rivas et al. [93] investigated the extracts and residual dietary fiber obtained from pomegranate peels using supercritical fluid extraction compared to the conventional method. The conventional method was conducted using an ethanol/water mixture (80:20, *v*/*v*) at 25 °C under agitation for 1 h, yielding 45.5% dietary fiber with a high galacturonic acid content of 64.8%. In contrast, supercritical CO_2_ extraction at 291 bar and 46.5 °C for 2.5 h resulted in a slightly lower yield (40.4%) and substantially lower galacturonic acid content of 30.4%, below the standard level of around 65% required to maintain functioning pectin. However, the supercritical CO_2_ method significantly outperformed the ethanol/water system in recovering phenolic compounds, yielding 4362 mg GAE/100 g. Residual dietary fiber exhibited an antioxidant activity of 6775 mg Trolox equivalents/100 g, as determined by the DPPH assay. In contrast, the conventional method resulted in a lower phenolic content of 2554 mg GAE/100 g and a residue with an antioxidant activity of 6160 mg Trolox equivalents/100 g. These results highlight the compromise between pectin quality and phenolic compound enrichment, suggesting that ethanol-based extraction is more favorable for obtaining pectin, whereas supercritical CO_2_ extraction is more effective for generating bioactivity-rich extracts and antioxidant-active residues, supporting its potential use in the development of nutraceutical and functional food ingredients.

### 4.4. Deep Eutectic Solvents

**Figure 6:**
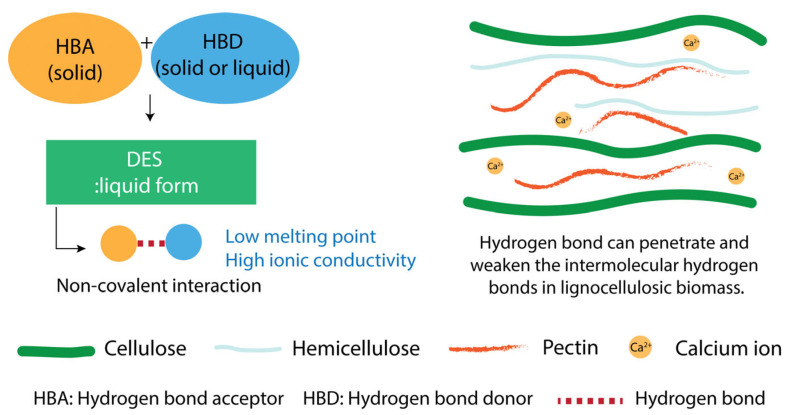
The effect of the DESs on pectin extraction.

Deep eutectic solvents (DESs) have gained significant attention as green and versatile alternatives for the extraction of pectin from plant biomass. These solvents, typically composed of a hydrogen bond acceptor (HBA) such as choline chloride and a hydrogen bond donor (HBD) such as organic acids or polyols, form eutectic mixtures with melting points significantly lower than those of the individual components [94,95]. Their unique properties, including low volatility, biodegradability, low toxicity, and tunable solvent characteristics, render them suitable for sustainable biorefinery applications [96,97,98]. The functionality of DESs can be achieved by modifying the HBD/HBA ratio and the chemical characteristics of each component to facilitate the selective solubilization of pectic polysaccharides while reducing the co-extraction of undesirable elements (Figure 6). Furthermore, DESs are often derived from renewable resources, are reusable, and align closely with the principles of green chemistry. Their ability to disrupt plant cell walls under mild temperature conditions without the use of mineral acids reduces both chemical waste and the degradation of the pectin structure [19,99].

Recent developments in pectin extraction research have highlighted the efficacy of DESs in enhancing both the yield and structural integrity of pectin obtained from plant materials (Table 3). The application of a ChCl/citric acid DES to *Averrhoa bilimbi* yielded 14.44% HMP with a DE of 54% and a highly branched sugar structure, which has been associated with enhanced antioxidant potential [100]. This suggests that DESs enable efficient pectin recovery and preserve structural features, such as neutral sugar side chains, that contribute to their bioactive properties. Conventional acid-based extraction using 0.05 M sulfuric acid from orange peel produced pectin with a DE of 72.92% and galacturonic acid content of 47.72%, yielding 12.64%. However, a significantly higher extraction efficiency was observed using a ChCl/formic acid (1:2), which yielded 46% HMP with a DE of 85.61% and a galacturonic acid content of 22.25%. Using MAE (360 W for 15 min) with the same DES formulation further improved the process efficiency, producing 40% pectin with a DE of 74.43% and galacturonic acid content of 29.5% [101]. These results indicate that the use of DESs with microwave energy may enhance pectin solubilization and reduce the thermal degradation of galacturonic acid but lower the DE. The extraction of pectin from mango peels further confirmed the advantages of the DESs. Conventional acid extraction (HCl, pH 2.5) produced 13.17% pectin with a DE of 65.55% and high MW (706 kDa). Utilization of ChCl/malic acid or betaine/citric acid led to a twofold increase in yield (30.01% and 27.62%, respectively), whereas the DE values were 83%, accompanied by elevated MWs (641–782 kDa) [102]. Hawthorn was extracted by combining UAE with mild acid (HCl, pH 1.5), resulting in 3.32% pectin with a high DE of 65.51%, galacturonic acid content of 83.66%, and MW of 309.32 kDa. Moreover, extraction using ChCl/urea (1:3) yielded 4.33% with a DE of 60.91%, galacturonic acid content of 88.93%, and MW of 156.73 kDa [103]. In contrast, ChCl/urea can reduce MW by promoting mild depolymerization during extraction. These results indicate that DESs with low acidity may preserve significant galacturonic acid content, DE, and MW when using HBA such as citric acid and malic acid. This highlights the potential of DESs to extract HMP with improved pectin purity while maintaining their structural integrity, which is often compromised by conventional acid hydrolysis. The ability to adjust the MW and DE by solvent selection and process optimization facilitates the precise design of pectins for particular applications, such as gelling agents, thickeners, and bioactive compounds. Moreover, DES-extracted pectins frequently exhibit superior biological functionalities, such as antioxidant and emulsifying capacities, which are attributed to the preservation of side chains and co-extracted phenolic compounds [104,105]. These results suggest that the ChCl/formic acid DES system yields a high extraction efficiency but results in a lower galacturonic acid content than sulfuric acid extraction, indicating its potential for the co-extraction of non-pectin compounds. In contrast, the ChCl/urea system has been shown to produce pectin with higher purity and galacturonic acid content, although it leads to reductions in both the DE and MW relative to conventional acid extraction. Betaine/citric acid and ChCl/malic acid systems have demonstrated the ability to enhance the pectin yield, DE, and MW. Notably, pectins extracted using DESs exhibited higher HG content than those in the RG-I region.

In summary, the different pectin extraction methods have distinct advantages and limitations (Table 4). Acid extraction is widely used to effectively solubilize protopectin; however, it can degrade pectin structures and negatively affect the environment. Similarly, alkali extraction is simple and useful for breaking pectin–protein complexes but may cause saponification or hydrolysis owing to its harsh conditions. Generally, EDTA is not readily biodegradable and can persist in aquatic environments, thus requiring proper wastewater treatment to comply with environmental regulations, which may increase operational costs. In contrast, ammonium oxalate and citric acid are biodegradable, naturally occurring, and pose lower environmental risks, making them more suitable for green and sustainable extraction processes. Chelating agents offer a high pectin yield while preserving the structural integrity under mild conditions; however, they often result in high ash content and require additional purification. The presence of divalent cations, such as calcium and magnesium, may enhance the ionic cross-linking of LMP, contributing to stronger gel formation. However, excessive ash content may also be associated with co-extracted impurities or reduced purity, potentially affecting the product consistency or sensory attributes in food applications. Subcritical fluids are recognized as environment-friendly solvents and are effective in extracting high-purity pectin, whereas supercritical fluid extraction is particularly efficient in preserving bioactive compounds. However, both techniques require expensive equipment and involve complex operational procedures. Nonetheless, these methods align more closely with green processing principles and may be more cost-effective in the long term, particularly when considering regulatory compliance and sustainability goals. DESs are emerging as green alternatives because of their tunable polarity and ability to retain the functional groups of pectin; however, their high viscosity and the need for careful optimization of the solvent composition can limit their extraction efficiency.

## 5. Functional Properties Related to the Structural Characteristics of Pectin

**Figure 7:**
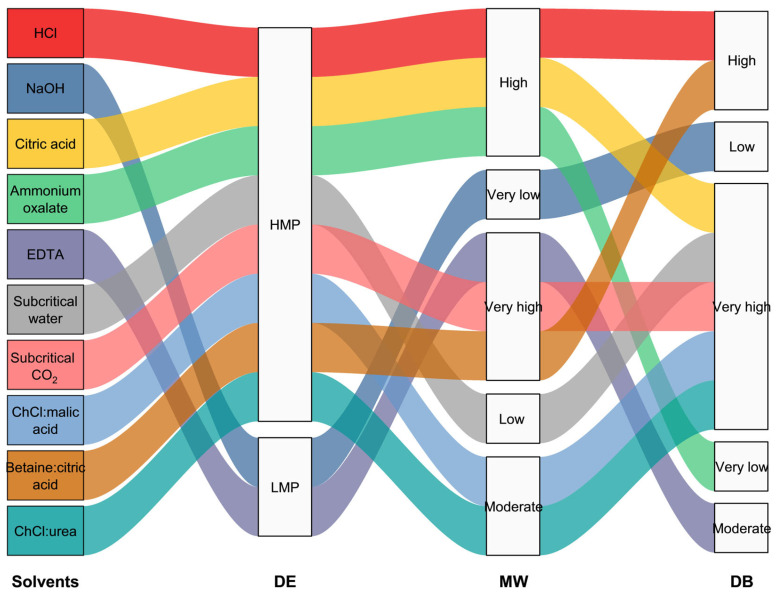
The alluvial plot illustrating the relationships between extraction solvents and structural attributes of pectin, based on data summarized in Table 1, Table 2, Table 3 and Table 4.

Pectin extraction using alternative solvents such as chelating agents, subcritical fluids, supercritical fluids, and DESs has gained increasing attention because of its ability to preserve structural integrity and enable environmentally friendly processing. This review summarizes the relationships between extraction solvents and the structural attributes of pectin, which are illustrated using an alluvial plot generated using OriginPro 2024 (OriginLab Corporation, Northampton, MA, USA) (Figure 7). This alluvial plot shows the relationships between various extraction solvents and the resulting structural attributes of pectin, including DE, MW, and DB. This visual representation enabled a qualitative comparison of the impact of different solvents on the pectin structure. The classification of each characteristic was interpreted and synthesized based on the experimental findings reported in Table 1, Table 2 and Table 3. These green extraction methods significantly influenced critical structural parameters, including DE, MW, and branching, depending on the type of solvent used. Modification of these structural features directly affects the functional properties of pectin, particularly its gelling ability, emulsifying capacity, water-holding ability, and biological activity. A clear understanding of the structure–function relationships of pectins obtained through these methods is essential for tailoring their functionalities to meet specific demands in food, pharmaceutical, and nutraceutical applications.

### 5.1. Structural Influence on Physicochemical Properties

The physicochemical properties of pectin, particularly its gelling characteristics, viscosity, emulsion stability, and water-holding capacity, are strongly influenced by its structural attributes. The development of pectin for various uses in food technology and other industries requires knowledge of these interactions. LMPs, which are typically derived from pectins extracted using alkaline solutions or chelating agents such as EDTA (Figure 7), can gel in the presence of calcium ions. This gelation occurs through the “egg-box” mechanism, where calcium ions facilitate cross-linking between neighboring pectin chains [106]. Unlike HMPs, LMPs do not require high sugar concentrations for gelation, highlighting their potential use as stabilizers or thickeners in low-sugar food products [107]. Moreover, LMPs have been reported to improve the texture and firmness of low-fat yogurt by interacting with the casein network, thereby enhancing the structural integrity of the gel matrix [108]. Cui et al. [109] reported that LMPs could increase the water-holding capacity and viscoelasticity of gluten protein networks, further supporting their functional roles in protein-rich systems. These findings suggest that LMPs possess a greater potential to interact with proteins than HMPs, making them suitable for various applications in dairy and bakery formulations, where protein–polysaccharide interactions are critical to product quality.

On the other hand, HMPs are typically produced through conventional acid extraction methods, although they can also be obtained using alternative, more environmentally sustainable solvents. The HMPs exhibited enhanced gelling properties and formed gels under acidic conditions at high sugar concentrations. The gelation mechanism involves the formation of junction zones, wherein methyl ester groups on adjacent pectin chains interact through hydrophobic associations and hydrogen bonding, leading to the development of a three-dimensional network capable of entrapping water [110]. Vithanage et al. [106] reported that HMP gels exhibit superior mechanical strength and elasticity compared with those formed by LMPs, primarily because of their higher DE, which facilitates stronger hydrophobic interactions and hydrogen bonding. Byun et al. [111] reported that polymer films fabricated from HMP exhibited greater burst strength, higher extensibility, and lower deformability than those fabricated from LMP. Nevertheless, HMPs have been reported to enhance their aggregation at the oil–water interface and improve their emulsifying properties owing to the hydrophobic nature of the methyl ester groups [112,113]. These properties make HMP appropriate for use in products that require firm gel textures, and they function effectively as emulsifiers.

The MW is another critical determinant of pectin functionality. Pectins extracted using EDTA, subcritical CO_2_, and a mixture of betaine and citric acid exhibited significantly higher MW than those obtained using conventional methods. Pectins with higher MW, typically ranging from 20 to 400 kDa, demonstrate enhanced polymer strength and viscosity. These pectins produce an extensive network that effectively captures water, improving the viscosity of the solution and enhancing its ability to form a stable gel [35,114]. Moreover, the structural complexity introduced by the sugar branches plays a significant role in defining the emulsion properties of pectin. Neutral sugar branches can enhance the emulsifying efficiency of pectin, thus influencing the stability and texture of the emulsions [13]. High-MW pectins facilitate the stabilization of emulsion droplets by increasing the thickness of the adsorbed layer, thereby preventing their combination and improving the overall emulsion stability [115]. Furthermore, RG-I-rich pectin protects emulsified oils from oxidation far better than high-DE citrus pectin. After 30 days, emulsions stabilized with RG I-rich pectin showed significantly lower lipid oxidation than those stabilized with citrus pectin, indicating delayed lipid peroxidation [116]. These structural characteristics highlight the importance of optimizing the molecular features of pectin to meet its specific functional requirements. In summary, the high DE, large MW, and structural complexity due to sugar branches contribute to the broad functionality of pectin, enhancing its gel strength, emulsion stability, and water-holding capacity, thereby expanding its potential applications.

### 5.2. Structural Influence on Biological Properties

The biological properties of pectin, including its prebiotic effects, immunomodulatory potential, and moisture retention ability, are significantly influenced by its structural characteristics. Previous studies have shown that low-MW LMPs exhibit enhanced antioxidant activity. This relationship is primarily attributed to the increased availability of free carboxyl groups and smaller molecular fragments, which scavenge free radicals more effectively [117,118]. In addition, RG-I-rich LMP extracted from watermelon rind showed significantly higher 2,2-diphenyl-1-picrylhydrazyl and hydroxyl radical scavenging activities than standard citrus pectin [116]. Xu et al. [119] reported that branched RG-I regions appear to confer additional antioxidant power, partly by entrapping radicals/oxidants within the gel network and providing additional hydroxyl and phenolic groups for radical quenching. In addition to their antioxidant activity, LMPs enriched in RG-I regions have demonstrated notable anti-inflammatory and immunomodulatory properties. Wu et al. [120] reported that purified RG-I pectin fragments can effectively attenuate inflammatory responses in immune cells by significantly reducing the production of nitric oxide and reactive oxygen species, as well as downregulating the expression of key pro-inflammatory cytokines, including IL-1β, IL-6, and TNF-α. In addition, animal models have shown that HMPs exert anti-inflammatory effects by mitigating inflammation associated with metabolic and gastrointestinal disorders [121]. Moreover, modified pectin with a high RG-I content and low MW has been associated with enhanced anticancer activity, particularly through the induction of apoptosis-like cell death in colon cancer cells. This structural configuration facilitates bioactive interactions with cancer cell membranes and signaling pathways involved in programmed cell death [121]. Moreover, the highly branched regions of pectin stimulate mucin secretion, thereby playing a protective role in the gut lining [122]. As illustrated in Figure 7, such properties were notably observed in pectin extracted using alternative solvents, which promoted partial depolymerization while preserving key bioactive regions such as RG-I. These findings suggest that specific extraction methods not only influence the physicochemical properties of pectin but also critically affect its bioactivity.

A previous study found that LMPs, often obtained from alkaline and EDTA extracts, are more readily degraded by the gut microbiota than HMP, thus resulting in elevated levels of short-chain fatty acids and several metabolites. In contrast, HMP, commonly obtained from alternative solvents, induces pronounced shifts in the gut microbial community, notably increasing the abundance of *Bacteroides* and reducing overall microbial diversity. These HMP-induced microbial alterations are associated with significant changes in immune cell infiltration within the lamina propria of the small intestine. Furthermore, HMP administration has been linked to reduced adipose tissue inflammation and lower levels of circulating pro-inflammatory mediators [123]. In addition, Ren et al. [124] reported that pectin has potential as an antidiabetic agent; however, different pectin structures have been found to exert distinct mechanisms in the management of diabetes. LMPs with low DE and high RG-I regions are more readily fermented in the colon, producing short-chain fatty acids such as acetate and propionate, which enhance insulin sensitivity and glucose metabolism. These pectins also modulate the gut microbiota to favor antidiabetic profiles. HMPs with higher DE and MW tend to have stronger gelling properties and slower fermentability, contributing to delayed gastric emptying and reduced postprandial glucose spikes, thus aiding glycemic control. In addition, a previous study found that pectins with a high DE (70%) and high MW derived from citrus and apple sources were more effective at lowering LDL cholesterol, thus achieving a reduction of 6–10% in humans [125]. This suggests that both high DE and MW, along with the pectin source, play crucial roles in the cholesterol-lowering effect. This suggests that both high DE and MW, along with the pectin source, play crucial roles in the cholesterol-lowering effect. RG-I-rich pectin has been associated with immunomodulatory effects, including the reduction of inflammation-related insulin resistance, and has shown promise in regulating adipokines and inflammatory cytokines involved in the pathogenesis of diabetes. In summary, specific structural features of pectin are critical determinants of its biological activity. These attributes influence the immunomodulatory effects, anticancer potential, prebiotic functions, antidiabetic mechanisms, and overall contribution to physiological health. Therefore, optimizing the pectin extraction and structural modification processes is essential for enhancing its targeted bioactivity and therapeutic potential.

## 6. Conclusions

The development of non-conventional extraction methods has led to significant advancements in the sustainable recovery of structurally intact and functionally diverse pectin. Techniques employing chelating agents, subcritical fluids, and DESs have demonstrated the ability to preserve key structural features, such as high MW, DE, and branched neutral sugar chains, which are critical determinants of gelling, emulsifying, and bioactive properties. Compared with traditional acid or alkali extractions, these methods offer enhanced selectivity and reduced degradation, aligned with green processing principles. Pectin obtained from ammonium oxalate and citric acid extraction generally retains a high DE and MW, making it suitable for conventional gelling applications such as firm-textured jams and jellies. In contrast, alkaline extraction and EDTA-based methods yield low-DE pectin (LMP), which undergoes gelation in the presence of calcium ions and is better suited for low-sugar food applications and functional dairy or bakery products, particularly because of its protein-interactive capacity. Extraction using subcritical water and DESs often results in pectins with lower MW but enhanced bioactivities, such as antioxidant and anticancer properties, especially when enriched in the RG-I regions. They are promising candidates for application in nutraceutical formulations, moisture retention systems, and prebiotics because of their fermentability and immunomodulatory potential. Future research should aim to refine these techniques, integrate renewable cosolvent systems, and establish standardized protocols for consistent quality. Such advancements will ensure that pectin extraction evolves toward environmentally responsible, high-performance applications in emerging bio-based industries.

## Figures and Tables

**Table 2:** An overview of the yield and physicochemical properties of pectin extracted using subcritical fluids.

Materials	Solvents	Extraction Conditions	Yield	Product Structures				Functionality	Ref.
				GalA (%)	DE (%)	MW (kDa)	Branching		
Citrus peel	Subcritical water	SLR 1:30 at 120 °C for 5 min	22.0	68.9	74.7	69.5	Ara 3.10%, Gal 2.52%	Higher anti-tumor	[23]
Apple pomace		SLR 1:30 150 °C for 5 min	16.7	40.1	86.0	53.4	Ara 2.33%, Gal 4.58%	Higher antioxidant	
Yuzu flavedo	Subcritical water	Hot compressed water at 160 °C with a pressure of 20 MPa	0.95	-	-	-	-	-	[51]
	Subcritical water + 0.4% SHMP		5.17	-	-	-	-		
	Subcritical water + 0.4% SHMP + 50 mM HCl		15.6	-	-	-	-		
Satsuma mandarin peel	* HCl, pH 2.5	SLR 1:25, at 90 °C for 90 min	10.4	63	75	65	High HG region	-	[77]
	Pressurized CO_2_	SLR 1:25 under 1 MPa at 90 °C for 90 min	3.8	61	>90	85	Rich in Ara and Gal	-	
Satsuma mandarin peel	* HCl, pH 1.4	Extraction in water bath at 85 °C for 70 min	16	76.6	71.4	1871	-	Emulsion	[83]
	Citric acid, pH 1.4		18	84.1	69.2	1674	-		
	High hydrostatic pressure	SLR 1:50 in citric acid solution pH 1.4, treated at 500 MPa for 10 min	19	77.0	67.7	1201	-		
Cocoa pod husk	Citric acid, pH 3	Extraction at 95 °C for 10 min	3.20	AUA 17.5%	29.8	EW 1429.5	-	-	[84]
	Subcritical water	SLR 1:15 extracted at 120 °C for 10 min	6.58	AUA 66.5%	37.9	EW 486.3	-		
Sugar beet pulp	* Acid extraction pH 1	Extraction at 90 °C for 4 h	20.8	68.2	57.0	102	-	-	[85]
	Subcritical water	SLR 1:30 130 °C for 20 min	20.7	73.0	84.2	23.5	-		
Unripe papaya	* Nitric acid	SLR 1:75 extracted at 80 °C 30 min	14.9	38.0	-	-	HG 27.7%, RG-I 55.9%	-	[86]
	Citric acid		5.90	31.2	-	127	HG 22.2%, RG-I 56.1%		
	Pressurized H_2_O + citric acid	SLR 1:75 40 MPa, 80 °C 30 min	4.60	41.1	-	68	HG 25.5%, RG-I 63.6%		
	Pressurized CO_2_ + H_2_O+ citric acid		16.8	51.2	-	149	HG 37.4%, RG-I 64.0%		

* Conventional solvent; AUA, Anhydroronic acid; GalA: Galacturonic acid; SLR: Solid–liquid ratio (g/mL); DB: Degree of branching; EW: Equivalent weight.

**Table 3:** An overview of the yield and physicochemical properties of pectin extracted using DESs.

Materials	Solvents	Extraction Conditions	Yield	Products Structure				Functionality	Ref.
				GalA (%)	DE (%)	MW (kDa)	Branching		
*Averrhoa bilimbi* (starfruit)	ChCl/citric acid, (1:1)	DES 3.74% at 80 °C for 2.5 h	14.4		54	-	highly branched sugar	Antioxidant	[100]
Orange peel	0.05 M sulfuric acid, pH 1.14	Incubated at 80 °C for 60 min	12.6	47.7	72.9	-	-	-	[101]
	ChCl/formic acid, (1:2)	8% DES, maceration at 90 °C, 60 min	46	22.3	85.6	-	-	-	
		8% DES, 360 W for 15 min using microwave	40	29.5	74.4	-	-	-	
Mango peel	* HCl, pH 2.5	SLR 1:40 at 90 °C for 2 h	13.2	61.5	65.6	706	HG 59.4%, RG-I 18.2%	-	[102]
	ChCl/malic acid	SLR 1:30 at 90 °C for 2 h	30.0	65.9	87.1	641	HG 63.56%, RG-I 19.25%	-	
	Betaine:citric acid		27.6	68.1	83.4	782	HG 66.3%, RG-I 18.6%		
Hawthorn	* HCl, pH 1.5	SLR 1:20 at 90 °C for 90 min	3.07	78.1	63.5	275	HG 94.0%, RG-I 5.98%	-	[103]
		SLR 1:15 UAE 70 °C for 40 min	3.32	83.7	65.5	309	HG 94.73%, RG-I 5.27%	-	
	ChCl/urea, (1:3)	SLR 1:30 at 80 °C for 60 min	4.33	88.9	60.9	157	HG 93.8%, RG-I 6.25%	-	

* Conventional solvents: AUA: Anhydrouronic acid; GalA: Galacturonic acid; SLR: Solid–liquid ratio (g/mL); DB: Degree of branching.

**Table 4:** A comparison of different pectin extraction methods.

Extraction Methods	Advantages	Disadvantages
Acid extraction	- High yield - Simple and low-cost	- Harsh conditions can degrade neutral sugars - High environmental burden from acid waste
Alkali extraction	- Effective in extracting pectin from hard tissues - Low cost	- Leads to de-esterification and degradation - Alters functional properties - Generates alkaline waste
Chelating agents	- Preserves side chains and DE - Gentle on pectin structure	- High ash content - Need additional purification steps
Subcritical fluids	- Environmentally friendly - Retain bioactivity	- Requires high-pressure equipment - Limited scalability
Supercritical fluids	- Selective extraction of bioactive compounds - Solvent-free	- High equipment and operation cost - Low pectin yield
DESs	- Tunable solvent properties - Preserve bioactivity	- Need optimization of HBA/HBD ratio - Residual solvent may remain in the product

## Data Availability

No new data were created or analyzed in this study. Data sharing is not applicable to this article.

## Abbreviations

The following abbreviations are used in this manuscript:

AUA	Anhydrouronic acid
CDTA	Cyclohexane diamine tetraacetic acid
DB	Degree of branching
DE	Degree of esterification
DES	Deep eutectic solvent
EAE	Enzyme-assisted extraction
EDTA	Ethylenediaminetetraacetic acid
EW	Equivalent weight
GalA	Galacturonic acid
HBA	Hydrogen bond acceptor
HBD	Hydrogen bond donor
HG	Homogalacturonan
HMP	High-methoxyl pectin
LMP	Low-methoxyl pectin
MAE	Microwave-assisted extraction
MW	Molecular weight
SHMP	Sodium hexametaphosphate
SLR	Solid–liquid ratio
UAE	Ultrasound-assisted extraction
XGA	Xylogalacturonan
